# Synthesis of Amphiphilic Block Copolymers Containing Chiral Polythiophene Chains and Their Micelle Formation and Chiroptical Properties

**DOI:** 10.3390/polym10070718

**Published:** 2018-06-30

**Authors:** Daisuke Hirose, Satoru Nozaki, Shigeyoshi Kanoh, Katsuhiro Maeda

**Affiliations:** 1 Graduate School of Natural Science and Technology, Kanazawa University, Kakuma-machi, Kanazawa 920-1192, Japan; dhirose@se.kanazawa-u.ac.jp (D.H.); satoru3959@yahoo.co.jp (S.N.); kanoh@se.kanazawa-u.ac.jp (S.K.); 2 Nano Life Science Institute (WPI-NanoLSI), Kanazawa University, Kakuma-machi, Kanazawa 920-1192, Japan

**Keywords:** polythiophene, chiral aggregate, circular dichroism, micelle, crosslinking

## Abstract

Amphiphilic block copolymers consisting of hydrophobic regioregular head-to-tail (HT) chiral ((*S*)-poly-**1a**-*b*-poly-**3**) or achiral (poly-**1b**-*b*-poly-**3**) polythiophene chains and a hydrophilic poly(acrylic acid) chain were synthesized. (*S*)-Poly-**1a**-*b*-poly-**3** with a chiral polythiophene block formed a micelle in water that exhibited a characteristic induced circular dichroism (ICD) in the π–π* transition region due to the formation of supramolecular π-stacked chiral aggregates of the chiral polythiophene blocks in the core. These aggregates were stable, showing no precipitation for more than 5 days. Micelles consisting of chiral (*S*)-poly-**1a**-*b*-poly-**3** and achiral poly-**1b**-*b*-poly-**3** showed negative nonlinear effects on supramolecular chiral aggregate formation in the core. Chiral polythiophene aggregates formed in (*S*)-poly-**1a**-*b*-poly-**3** micelle cores were stabilized by the crosslinking of poly(acrylic acid) blocks with diamines in the shell. The ICD intensity of the (*S*)-poly-**1a**-*b*-poly-**3** micelle after shell crosslinking showed almost no change with temperature, while that before shell crosslinking decreased with increasing temperature.

## 1. Introduction

Regioregular (head-to-tail, HT) polythiophenes (PTs) bearing an optically active substituent are known to form supramolecular chiral aggregates in poor solvents or films. These molecules show an induced circular dichroism (CD) in the π–π* transition region that is attributed to the PT main chain, but show no induced CD in the same region in a good solvent [1,2,3,4,5,6,7]. These chiral supramolecular aggregates have attracted considerable interest owing to their promising applications, for example, in enantioselective catalysts, adsorbents, sensors, and electrodes [1,2,3]. However, the stability of chiral aggregates produced by intermolecular π-stacking interactions is highly dependent on external conditions, such as the solvent, temperature, and concentration. These well-organized structures cannot be maintained for long periods due to precipitation caused by excessive aggregation.

In contrast, amphiphilic block copolymers consisting of a hydrophobic chain and hydrophilic chain are known to form polymer micelles or vesicles in water. These polymer micelles are predicted to have applications as nanoreactors and drug delivery systems through the use of the hydrophobic cores as a closed molecular space [8]. Furthermore, dynamic polymer micelles can be easily transformed into stable robust nanoparticles by crosslinking the shell using a well-established procedure [9,10,11].

**Scheme 1:**
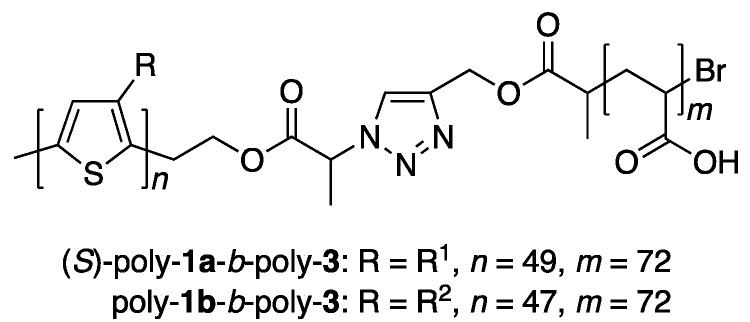
Structures of (*S*)-poly-**1a**-*b*-poly-**3** and poly-**1b**-*b*-poly-**3**.

In this study, we synthesized amphiphilic block copolymers consisting of a hydrophobic chiral or achiral PT chain and a hydrophilic poly(acrylic acid) chain ((*S*)-poly-**1a**-*b*-poly-**3** or poly-**1b**-*b*-poly-**3**, respectively) (Scheme 1). The chiroptical properties of their micelles formed in an aqueous solution were investigated using circular dichroism (CD) and absorption spectroscopy to determine whether the PT chains formed chiral supramolecular aggregates effectively in the micelle core. We also investigated the effect of shell crosslinking on the stability of the chiral aggregates formed in the micelle core.

## 2. Materials and Methods

### 2.1. Reagents and Materials

Anhydrous toluene, dichloromethane, and diethyl ether were obtained from Kanto Kagaku (Tokyo, Japan). Triethylamine (TEA) was dried over KOH pellets and distilled from KOH under nitrogen. These solvents were stored under nitrogen. Anhydrous tetrahydrofuran (THF), 3-bromothiophene, *tert*-butyl acrylate, trifluoroacetic acid, [1,3-bis(diphenylphosphino)propane]dichloronickel(II) (Ni(dppp)Cl_2_), *N*,*N*,*N*′,*N*′′,*N*′′-pentamethyldiethylenetriamine (PMDETA), and palladium on activated carbon (Pd/C, 10 wt %) were purchased from Wako Pure Chemical Industries (Osaka, Japan). (*S*)-(+)-Citronellyl bromide, 2-bromopropionyl bromide, 1-[3-(dimethylamino)propyl]-3-ethylcarbodiimide methiodide, *n*-butylmagnesium chloride (2.0 M in THF), vinylmagnesium bromide (1.0 M in THF), 9-borabicyclo[3,3,1]nonane (9-BBN) (0.5 M in THF), and tetrabutylammonium fluoride (TBAF) (1.0 M in THF) were obtained from Sigma-Aldrich (Milwaukee, WI, USA). 1,2-Dibromoethane, azidotrimethylsilane (Me_3_SiN_3_), 2,2′-(ethylenedioxy)bis(ethylamine), and propargyl alcohol were purchased from Tokyo Chemical Industry (TCI, Tokyo, Japan). 1-Bromohexane and ethyl 2-bromopropionate were obtained from Kanto Kagaku (Tokyo, Japan). Prop-2-yn-1-yl 2-bromopropanoate [12], (*S*)-2,5-dibromo-3-(3,7-dimethyloctyl)thiophene ((*S*)-**1a**) [13], and 2,5-dibromo-3-hexylthiophene (**1b**) [14] were prepared according to previously reported methods.

### 2.2. Instruments

NMR spectra were recorded on an NM-LA400FT (JEOL, Tokyo, Japan; 400 MHz for ^1^H) or EX270 (JEOL; 270 MHz for ^1^H) spectrometer in CDCl_3_ using TMS as the internal standard. IR spectra were recorded with a JASCO Fourier Transform IR-460 spectrophotometer (Hachioji, Japan). Absorption and CD spectra were measured in a 1.0 mm quartz cell on a JASCO V-570 spectrophotometer and a JASCO J-725 spectropolarimeter, respectively. The temperature was controlled using a JASCO PTC-348WI apparatus. Size exclusion chromatography (SEC) measurements were performed with a JASCO PU-2080 liquid chromatograph equipped with a UV–vis (Jasco UV-970) detector at 40 °C. The temperature was controlled using a JASCO CO-965 column oven. A Tosoh TSKgel MultiporeH_XL_-M column (30 cm; Tokyo, Japan) was used for SEC measurements using THF as the eluent at a flow rate of 1.0 mL/min. Molecular weight calibration curves were obtained using polystyrene standards (Tosoh). Dynamic light scattering (DLS) measurements were recorded with a Beckman Coulter N5 instrument (Brea, CA, USA).

### 2.3. Synthesis

#### 2.3.1. Synthesis of Amphiphilic Block Copolymers

**Scheme 2:**
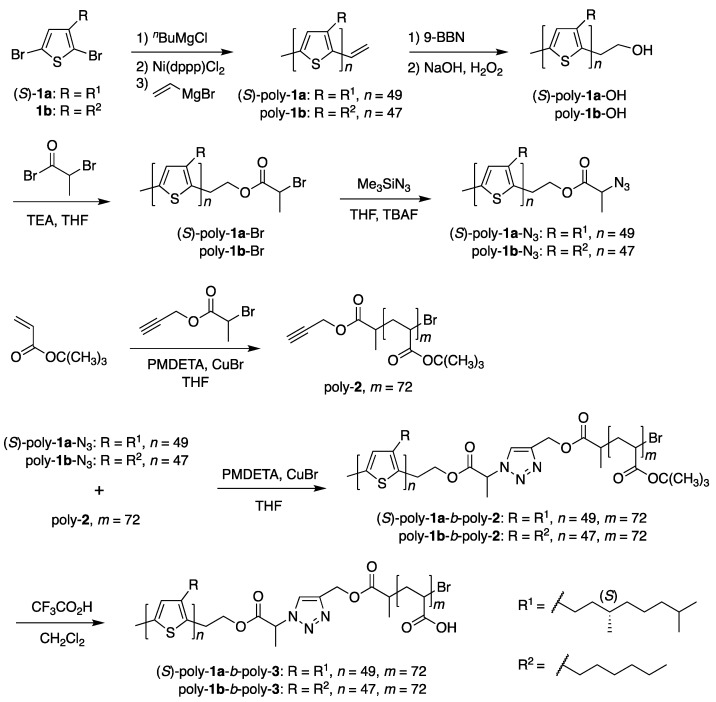
Synthesis of amphiphilic block copolymers (*S*)-poly-**1a**-*b*-poly-**3** and poly-**1b**-*b*-poly-**3**.

Amphiphilic block copolymers (*S*)-poly-**1a**-*b*-poly-**3** and poly-**1b**-*b*-poly-**3** were synthesized using the route shown in Scheme 2.

*Synthesis of (S)-poly-***1a***:* (*S*)-**1a** was polymerized according to the procedure of McCullough [15,16]. Under dry nitrogen, 2 M *n-*butylmagnesium chloride in THF was slowly added to the THF solution (10 mL) of (*S*)-**1a** (2.0 g, 5.3 mmol) using a syringe. After stirring the mixture for 2.5 h at room temperature (rt), a suspension of Ni(dppp)Cl_2_ (0.061 g, 0.11 mmol) in THF (38 mL) was added, followed by 1 M vinylmagnesium bromide in THF (1.5 mL, 1.5 mmol), and the resulting mixture was stirred for 10 min. The solvent was then evaporated, the mixture poured into a large amount of methanol, and the resulting polymer collected by centrifugation, washed with methanol, and dried in vacuo. A Soxhlet purification was performed using methanol, hexane, and chloroform sequentially to obtain (*S*)*-*poly-**1a** as a green solid (0.53 g, 44% yield). ^1^H NMR (270 MHz, CDCl_3_, rt): *δ* 6.97 (s, 1H, Ar–*H*), 2.68–2.92 (m, Ar–C*H*_2_), 1.12–1.82 (m, 10H, 4C*H*_2_ and 2C*H*), 0.98 (d, *J* = 5.9 Hz, 3H, C*H*_3_), 0.86 (d, *J* = 6.5 Hz, 6H, 2C*H*_3_). From the ^1^H NMR spectrum (Figure S1), the degree of polymerization (DP) and the number average molecular weight (*M*_n_) were determined as 49 and 1.1 × 10^4^, respectively, based on the integral ratio of the proton signal on the thiophene ring in the repeat unit to the proton signals of the vinyl groups at the ends. The *M*_n_ and its distribution (*M*_w_/*M*_n_) were estimated to be 1.3 × 10^4^ and 1.2, respectively, by SEC using polystyrene standards with THF as the eluent at 40 °C.

*Synthesis of poly-***1b***:* poly-**1b** was synthesized using the same method as (*S*)*-*poly-**1a** but from **1b**. ^1^H NMR (270 MHz, CDCl_3_, rt): *δ* 6.98 (s, 1H, Ar–*H*), 2.80 (t, *J* = 7.6 Hz, 2H, Ar–C*H*_2_), 1.65–1.73 (m, 2H, Ar–CH_2_C*H*_2_), 1.25–1.50 (m, 6H, 3C*H*_2_), 0.91 (t, *J* = 6.7 Hz, 3H, C*H*_3_). The DP and *M*_n_ were determined to be 47 and 7.8 × 10^3^, respectively, by ^1^H NMR analysis. The *M*_n_ and *M*_w_/*M*_n_ estimated using SEC were 1.1 × 10^4^ and 1.4, respectively.

*Synthesis of (S)-poly-***1a***-OH:* Under dry nitrogen, 0.5 M 9-BBN in THF (11.3 mL, 5.7 mmol) was slowly added to a THF solution (60 mL) of (*S*)-poly-**1a** (0.51 g, 0.046 mmol) using a syringe, and the mixture was stirred for 24 h at 40 °C. Next, 6 M NaOH (6.6 mL) was added at rt and the mixture was stirred for 15 min. Thirty percent H_2_O_2_ (6.6 mL) was then added and the mixture was stirred for 24 h at 40 °C. After evaporating the solvent, the mixture was poured into a large amount of methanol/water (1/1, *v/v*), and the resulting polymer was collected by centrifugation, washed with methanol/water (1/1, *v/v*), and dried in vacuo. A Soxhlet purification was performed using methanol to obtain (*S*)-poly-**1a**-OH as a green solid (0.50 g, 99% yield).

*Synthesis of (S)-poly-***1a***-Br:* Under dry nitrogen, TEA (4.5 mL, 32.2 mmol) was slowly added to the THF solution (48 mL) of (*S*)-poly-**1a**-OH (0.48 g, 0.048 mmol) with a syringe, and the mixture was stirred for 15 min at 40 °C. After the addition of 2-bromopropionyl bromide (3.6 mL, 34.0 mmol), the mixture was stirred for 24 h. After the solvent was evaporated, the mixture was poured into a large amount of methanol, and the resulting polymer was collected by centrifugation, washed with methanol, and dried in vacuo. A Soxhlet purification was performed using methanol to obtain (*S*)-poly-**1a**-Br as a green solid (0.51 g, quantitative yield).

*Synthesis of (S)-poly-***1a***-N_3_:* Under dry nitrogen, 1.0 M TBAF in THF (190 µL, 0.19 mmol) was slowly added to a THF solution (15 mL) of (*S*)-poly-**1a**-Br (0.21 g, 0.019 mmol) and Me_3_SiN_3_ (25 µL, 0.19 mmol) using a syringe, and the mixture was stirred for 48 h at rt. After evaporating the solvent, the mixture was poured into a large amount of methanol, and the resulting polymer was collected by centrifugation, washed with methanol, and dried in vacuo. A Soxhlet purification was performed using methanol to obtain (*S*)-poly-**1a**-N_3_ as a green solid (0.192 g, 92% yield).

*Synthesis of poly-***2***:* Poly-**2** was synthesized according to a literature method [17]. Prop-2-yn-1-yl 2-bromopropanoate (78 mg, 0.041 mmol), *tert*-butyl acrylate (6.0 mL, 40.8 mmol), toluene (6.0 mL), and CuBr (58.6 mg, 0.41 mmol) were placed in a dry Schlenk flask under an argon atmosphere. PMDETA (86 µL, 0.41 mmol) was added and the mixture was stirred for 9 h at 80 °C. The solution was extracted with chloroform and the organic layer was washed with water until the blue color of copper had completely disappeared and then dried over Na_2_SO_4_. After evaporating the solvent, the obtained product was dissolved in a small amount of methanol, poured into a large amount of methanol/water (7/3, *v/v*) at 0 °C, and the resultant precipitated polymer was washed with methanol/water (7:3, *v/v*) and dried in vacuo to afford poly-**2** as a white solid (2.19 g, 42% yield). ^1^H NMR (400 MHz, CDCl_3_, rt): *δ* 2.15–2.35 (br, 1H, CH of the polymer backbone), 1.74–1.90 (br, meso CH_2_ of the polymer backbone), 1.20–1.60 (br, *meso* and *racemo* CH_2_ of the polymer backbone), 1.20–1.50 (br, 3CH_3_). The DP and *M*_n_ were determined as 72 and 9.2 × 10^3^, respectively, by ^1^H NMR analysis based on the integral ratio of the proton signal of the methine group in the repeat unit to the proton signals of the methylene group next to the terminal ethynyl group. The *M*_n_ and *M*_w_/*M*_n_ were also estimated to be 9.1 × 10^3^ and 1.3, respectively, by SEC using polystyrene standards in THF as the eluent at 40 °C.

*Synthesis of (S)-poly-***1a***-b-poly-***2***:* In a dry Schlenk flask, CuBr (6.7 mg, 0.046 mmol) was added to a THF solution (2 mL) of (*S*)-poly-**1a**-N_3_ (0.050 g, 0.0046 mmol), poly-**2** (0.11 g, 0.012 mmol), and PMDETA (10 µL, 0.046 mmol) under dry nitrogen, and the mixture was stirred for 4 h at rt. After evaporating the solvent, the mixture was poured into a large amount of methanol, and the resulting polymer was collected by centrifugation, washed with methanol, and dried in vacuo to obtain (*S*)-poly-**1a**-*b*-poly-**2** as a purple solid (75 mg, 82% yield). IR (KBr, cm^–1^): 1727 (ν_C=O of ester_). ^1^H NMR (400 MHz, CDCl_3_, rt): *δ* 6.98 (s, 1H, Ar–H), 2.81 (br, 2H, Ar–CH_2_), 2.27 (br, 1H, CH of poly(acrylic acid)), 1.10–1.90 (m, 21H), 0.98 (d, 3H, CH_3_), 0.87 (d, 6H, 2CH_3_).

*Synthesis of (S)-poly-***1a***-b-poly-***3***:* Under dry nitrogen, trifluoroacetic acid (0.1 mL, 1.35 mmol) was added to a CHCl_3_/1,4-dioxane (5/2, *v/v*) solution (4.2 mL) of (*S*)-poly-**1a**-*b*-poly-**2** (0.061 g, 0.22 mmol), and the mixture was stirred for 24 h at 40 °C. After the solvent was evaporated, the mixture was washed with methanol and dried in vacuo to obtain (*S*)-poly-**1a**-*b*-poly-**3** as a black solid (43 mg, 88% yield). IR (KBr, cm^–1^): 1727 (ν_C=O of carboxylic acid_). ^1^H NMR (400 MHz, CDCl_3_, rt): *δ* 6.98 (s, 1H, Ar–H), 2.76 (br, 2H, Ar–CH_2_), 2.05 (br, 1H, CH of poly(acrylic acid)), 1.05–1.80 (m, 12H), 0.97 (d, 3H, CH_3_), 0.86 (d, 6H, 2CH_3_).

*Synthesis of poly-***1b***-b-poly-***3***:* Poly-**1b**-*b*-poly-**3** was synthesized using the same method as (*S*)-poly-**1a**-*b*-poly-**3** through the click coupling of poly-**1b** and poly-**2** followed by hydrolysis of the *tert*-butyl ester groups. IR (KBr, cm^–1^): 1727 (ν_C=O of carboxylic acid_). ^1^H NMR (400 MHz, CDCl_3_, rt): *δ* 6.98 (s, 1H, Ar–H), 2.81 (br, 2H, Ar–CH_2_), 2.05 (br, 1H, CH of poly(acrylic acid)), 1.10–1.90 (m, 10H), 0.92 (br, 3H, CH_3_).

#### 2.3.2. Crosslinking of (*S*)-Poly-**1a**-*b*-poly-**3** Micelles

To a THF solution (17 mL) of (*S*)-poly-**1a**-*b*-poly-**3** (5.0 mg, 22 µmol) was added water (17 mL) at a rate of 0.2 mL/min using a syringe pump. After removing THF in vacuo, 2,2′-(ethylenedioxy)bis(ethylamine) (829 µg, 5.6 µmol) in water (170 µL) was added slowly and the mixture was stirred for 1 h at rt. Next, an aqueous solution of 1-[3’-(dimethylamino)propyl]-3-ethylcarbodiimide methiodide (3.3 mg, 11 µmol, 3.3 mL) was added at a rate of 0.2 mL/min using a syringe pump. The resulting polymer was purified using a dialysis membrane (MWCO 3500) in water for 4 days. After removing the water by freeze drying, crosslinked micelles of (*S*)-poly-**1a**-*b*-poly-**3** were obtained.

## 3. Results and Discussion

### 3.1. Synthesis of Amphiphilic Block Copolymers

Regioregular HT chiral ((*S*)-poly-**1a**-N_3_) and achiral (poly-**1b**-N_3_) PTs possessing a terminal azide group were synthesized according to a literature method (Scheme 2) [15,16,17,18,19,20]. Vinyl-terminated PTs (*S*)-poly-**1a** and poly-**1b** were first synthesized from the corresponding 2,5-dibromothiophene derivatives using a Grignard metathesis method (GRIM) with *n*-butylmagnesium chloride, Ni(dppp)Cl_2_, and vinylmagnesium bromide as the end-capping reagent [15,16,18]. Based on the integral ratio of the signal attributed to the proton at the 4-position of the thiophene ring to that attributed to the terminal vinyl protons in the ^1^H NMR spectra of (*S*)-poly-**1a** and poly-**1b**, the average degree of polymerization (DP) and the number average molecular weight (*M*_n_) were determined as 49 and 1.1 × 10^4^ ((*S*)-poly-**1a**), and 47 and 7.8 × 10^3^ Da (poly-**1b**), respectively (Figure S1). The terminal vinyl groups of (*S*)-poly-**1a** and poly-**1b** were first converted into hydroxyethyl groups by hydroboration–oxidation ((*S*)-poly-**1a**-OH and poly-**1b**-OH) and the resulting hydroxy groups were then converted to bromoesters by reacting with 2-bromopropionyl bromide ((*S*)-poly-**1a**-Br and poly-**1b**-Br) [15,16,18]. The terminal bromide groups of (*S*)-poly-**1a**-Br and poly-**1b**-Br were further converted into azide groups by nucleophilic substitution with Me_3_SiN_3_ to give (*S*)-poly-**1a**-N_3_ and poly-**1b**-N_3_. Conversion of the terminal groups was confirmed in each step by ^1^H NMR spectroscopy (see Figures S1−S4).

Poly(*tert*-butyl acrylate) with a terminal ethynyl group (poly-**2**) was synthesized by the atom transfer radical polymerization (ATRP) of *tert*-butyl acrylate using copper(I) bromide as the catalyst and prop-2-yn-1-yl 2-bromopropanoate as the initiator in toluene according to a previously reported method (Scheme 2) [17,21,22]. The DP and *M*_n_ of poly-**2** were determined as 72 and 9.2 × 10^3^ Da, respectively, based on the integral ratio of the methine proton signal in the repeat unit at 2.2 ppm to the methylene proton signals next to the terminal ethynyl group at 4.65 ppm in the ^1^H NMR spectrum of poly-**2** (Figure S5).

**Figure 1:**
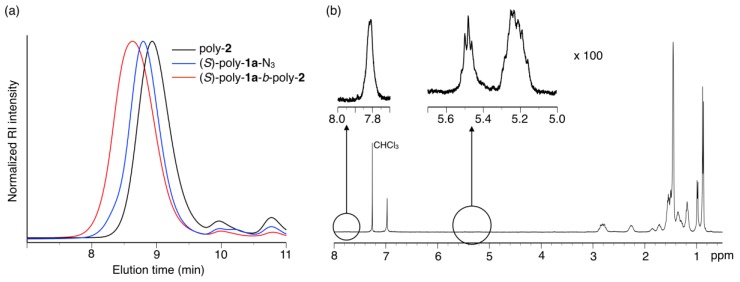
Results of the click reaction of (*S*)-poly-**1a**-N_3_ with poly-**2**: (**a**) size exclusion chromatography (SEC) traces of (*S*)-poly-**1a**-*b*-poly-**2**, poly-**2**, and (*S*)-poly-**1a**-N_3_ in tetrahydrofuran (THF) at 40 °C (PSt standard); (**b**) ^1^H NMR spectrum of (*S*)-poly-**1a**-*b*-poly-**2** in CDCl_3_ at rt.

Chiral (*S*)-poly-**1a**-N_3_ and poly-**2** were transformed into the corresponding block copolymer ((*S*)-poly-**1a**-*b*-poly-**2**) connected to a triazole ring by a Huisgen cycloaddition using CuBr and PMDETA as the catalyst [17,23,24,25]. Figure 1a shows SEC chromatograms of the obtained (*S*)-poly-**1a**-*b*-poly-**2** and precursor polymers (*S*)-poly-**1a**-N_3_ and poly-**2**). The peak of (*S*)-poly-**1a**-*b*-poly-**2** was shifted to a higher molecular weight than the peaks of both (*S*)-poly-**1a**-N_3_ and poly-**2**, while maintaining the unimodal shape. In the ^1^H NMR spectrum of (*S*)-poly-**1a**-*b*-poly-**2**, peaks attributed to the triazole ring were clearly observed at around 5.0–5.6 ppm and 7.7–7.9 ppm, suggesting that the block copolymer was successfully formed through a click reaction. (Figure 1b). Hydrolysis of the *tert-*butyl group of (*S*)-poly-**1a**-*b*-poly-**2** with trifluoroacetic acid afforded the corresponding amphiphilic block copolymer with a poly(acrylic acid) chain ((*S*)-poly-**1a**-*b*-poly-**3**). The hydrolysis of the *tert-*butyl group was confirmed as complete by the disappearance of the peak attributed to the *tert-*butyl group in the ^1^H NMR spectrum (Figure S6). Similarly, an amphiphilic block copolymer containing an achiral PT chain (poly-**1b**-*b*-poly-**3**) was prepared by the click reaction of poly-**1b** with poly-**2** followed by hydrolysis of the *tert-*butyl group with trifluoroacetic acid (Figures S7 and S8).

### 3.2. Chiroptical Properties

**Figure 2:**
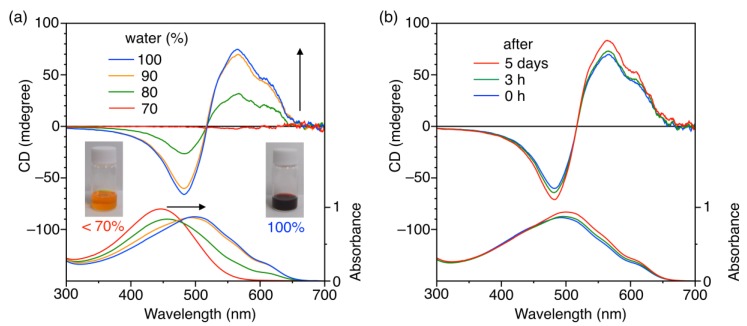
Circular dichroism (CD) and absorption spectra of (*S*)-poly-**1a**-*b*-poly-**3** in THF/water at rt: (**a**) effect of THF in water; (**b**) time-dependent change in water. [(*S*)-poly-**1a**-*b*-poly-**3**] = 0.5 mg/mL.

The amphiphilic block copolymer (*S*)-poly-**1a**-*b*-poly-**3** was expected to form micelles by adding water to a THF solution of (*S*)-poly-**1a**-*b*-poly-**3** at a flow rate of 0.1 mL/min using a syringe pump because (*S*)-poly-**1a**-*b*-poly-**3** is insoluble in water but soluble in THF. Figure 2a shows the CD and absorption spectra of (*S*)-poly-**1a**-*b*-poly-**3** in THF with increasing amounts of water. The CD spectrum of (*S*)-poly-**1a**-*b*-poly-**3** in THF showed almost no CD in the wavelength region of the PT backbone absorption, and similar spectra were observed at water contents of up to 70%. However, at a water content of 80%, the peak of the absorption spectrum was significantly redshifted, the solution color significantly changed from orange to purple, and an intense split-type positive Cotton effect was observed in the π–π* transition region of the PT main chain. At a water content of 90%, the absorption spectra of (*S*)-poly-**1a**-*b*-poly-**3** showed a further bathochromic shift with a clear isosbestic point at 477 nm and a further increase in CD intensity. The solution remained homogeneous, even after the removal of THF under reduced pressure, and the CD and absorption spectra in water were almost the same as those at a water content of 90%. These results indicated that the amphiphilic block copolymer (*S*)-poly-**1a**-*b*-poly-**3** formed micelles in water, with the hydrophobic PT chain forming a supramolecular chiral aggregate in the micelle cores. To investigate the size of the micelle comprising (*S*)-poly-**1a**-*b*-poly-**3**, DLS measurements were conducted in water and indicated the formation of near-monodisperse micelles with a hydrodynamic radius (*R*_h_) of approximately 50 nm (see Figure S9) [6].

Figure 2b shows the time-dependent CD and absorption spectral changes of (*S*)-poly-**1a**-*b*-poly-**3** in water. The CD intensity slightly increased and was accompanied by a slight redshift of the absorption peak after 5 days, and no precipitation was observed, even after 1 week. In contrast, chiral aggregates derived from chiral thiophene homopolymer ((*S*)-poly-**1a**) with only a hydrophobic chain in a poor solvent system (CHCl_3_/methanol (1/1, *v/v*)) at rt showed a gradual decrease in the CD intensity with time (see Figure S10), with the CD intensity decreasing by more than half the initial value after 24 h due to precipitation of the chiral aggregates. This demonstrates that inducing aggregate formation in the micelle core is a useful method for obtaining chiral supramolecular aggregates that are stable in solution for long periods.

**Figure 3:**
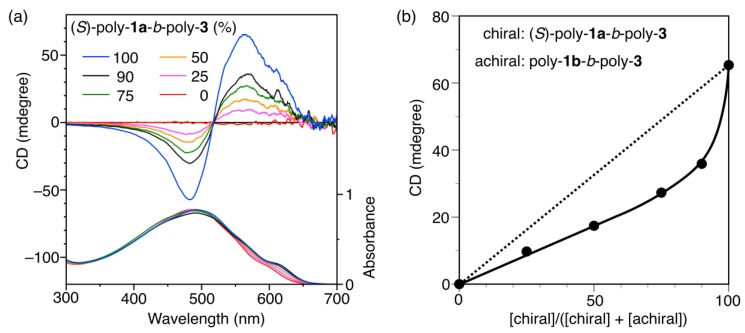
(**a**) CD and absorption spectra of aggregates consisting of (*S*)-poly-**1a**-*b*-poly-**3** and poly-**1b**-*b*-poly-**3**; (**b**) Plots of CD intensity at around 565 nm for aggregates consisting of (*S*)-poly-**1a**-*b*-poly-**3** and poly-**1b**-*b*-poly-**3** in THF/H_2_O (9:1, *v/v*) at rt.

The chiral amplification behavior of chiral aggregate formation of (*S*)-poly-**1a**-*b*-poly-**3** in the micelle core was also investigated. In this experiment, micelles were formed from mixtures of chiral (*S*)-poly-**1a**-*b*-poly-**3** and achiral poly-**1b**-*b*-poly-**3** at various feed ratios in THF/water (9:1, *v/v*) and CD and absorption spectra of these micelles were measured (Figure 3a). Figure 3b shows plots of the observed CD intensity at 565 nm against the feed ratio of chiral (*S*)-poly-**1a**-*b*-poly-**3** and a strong negative nonlinear relationship was observed [26], where the observed CD intensity was much lower than predicted by a linear relationship (dotted line in Figure 3b). This result suggested that cooperative interactions between the chiral PT chains played an important role in chiral aggregate formation in the micelle core. Similar negative nonlinear effects have been reported for the formation of chiral supramolecular aggregates of chiral PT chains in a poor solvent [27,28].

### 3.3. Micelle Stabilization through Crosslinking

**Figure 4:**
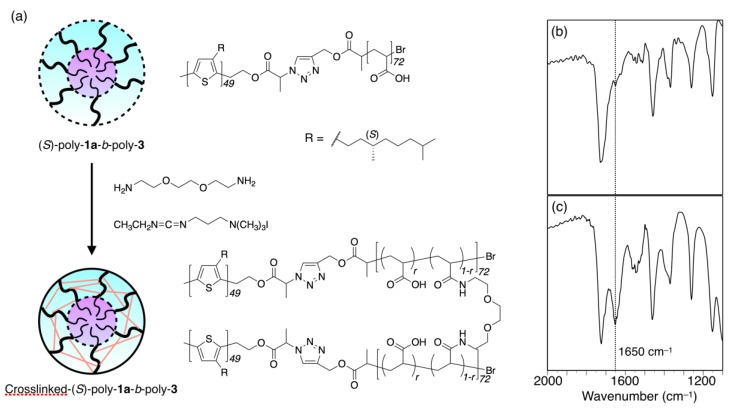
(**a**) Schematic illustration of shell crosslinking of micelles comprising (*S*)-poly-**1a**-*b*-poly-**3** and (**b**,**c**) IR spectra of (**b**) ((*S*)-poly-**1a**-*b*-poly-**3** and (**c**) crosslinked-(*S*)-poly-**1a**-*b*-poly-**3** at rt.

**Figure 5:**
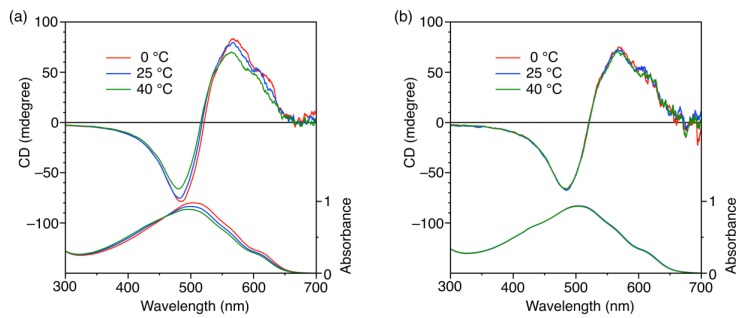
CD and absorption spectra of the micelles comprising (*S*)-poly-**1a**-*b*-poly-**3** (**a**) before and (**b**) after shell crosslinking in water at various temperatures.

The crosslinking of micelles in the shell domain has been reported to lead to the formation of robust nanoparticles known as shell-crosslinked micelles. We performed the shell crosslinking of micelles of (*S*)-poly-**1a**-*b*-poly-**3** to determine whether shell crosslinking could increase the stability of the internal chiral aggregate structure formed in the core domain of the micelles. Nominally 50% of the carboxylic acid groups in the poly(acrylic acid) shell of the micelles of (*S*)-poly-**1a**-*b*-poly-**3** were crosslinked using a water-soluble diamine, 2,2′-(ethylenedioxy)bis(ethylamine), as a crosslinker molecule and a water-soluble carbodiimide, 1-[3-(dimethylamino)propyl]-3-ethylcarbodiimide methiodide, as a condensing reagent, according to a reported method (Figure 4a) [11,29,30]. After the reaction and purification by dialysis, shell crosslinking through amide bond formation was confirmed by IR analysis, with new peaks attributed to the amide group observed at around 1650 cm^−1^ (amide I) and 1560 cm^–1^ (amide II) in the IR spectrum of the resulting micelle (Figure 4b). Figure 5 shows the CD and absorption spectra of (*S*)-poly-**1a**-*b*-poly-**3** in water at various temperatures before and after shell crosslinking. Before shell crosslinking, the CD intensity of the micelles decreased with increasing temperature (Figure 5a). However, after crosslinking, the CD and absorption spectra of the shell-crosslinked micelles showed almost no change in the CD intensity at any temperature (Figure 5b). This demonstrated that shell crosslinking of the micelle stabilized the chiral supramolecular aggregates of the PT chains formed in the core.

## 4. Conclusions

In conclusion, amphiphilic block copolymers consisting of hydrophobic regioregular chiral or achiral PT chains and a hydrophilic poly(acrylic acid) chain were successfully synthesized using a macromolecular click reaction. The chiroptical properties of micelles of the amphiphilic block copolymers containing the chiral PT block were investigated to evaluate the chiral aggregate formation behavior of the PT chains in micelles. Micelles consisting of poly(acrylic acid) as the shell and optically active PT as the core were generated by self-assembly in water, and chiral supramolecular aggregates were formed in the core that were stable in aqueous solution for long periods without precipitation. Chiral aggregates consisting of chiral (*S*)-poly-**1a**-*b*-poly-**3** and achiral poly-**1b**-*b*-poly-**3** formed in the micelle core showed a negative nonlinear relationship between the observed CD intensity and the feed ratio of the chiral unit. Chiral supramolecular aggregates of PT chains formed in the core were highly stabilized by shell crosslinking of the micelles, as evidenced by the CD intensity showing almost no change with increasing temperature. The shell-crosslinked robust nanoparticles possessing chiral supramolecular aggregates in the hydrophobic core could potentially be used as enantioselective sensors and chiral catalysts for asymmetric synthesis. Studies of such applications are currently in progress.

## Supplementary Materials

The following are available online at http://www.mdpi.com/2073-4360/10/7/718/s1, Figure S1: ^1^H NMR spectrum of (*S*)-poly-**1a**, Figure S2: ^1^H NMR spectrum of (*S*)-poly-**1a**-OH, Figure S3: ^1^H NMR spectrum of (*S*)-poly-**1a**-Br, Figure S4: ^1^H NMR spectrum of (*S*)-poly-**1a**-N_3_, Figure S5: ^1^H NMR spectrum of poly-**2**, Figure S6: ^1^H NMR spectrum of (*S*)-poly-**1a**-*b*-poly-**3**, Figure S7: ^1^H NMR spectrum of poly-**1b**-*b*-poly-**2**, Figure S8: ^1^H NMR spectrum of poly-**1b**-*b*-poly-**3**, Figure S9: DLS data of the micelles comprising (*S*)-poly-**1a**-*b*-poly-**3**, Figure S10: Time-dependent CD intensity change of (*S*)-poly-**1a** in CHCl_3_/MeOH (1/1, *v*/*v*).

## References

[1] Langeveld-Voss B.M.W., Janssen R.A.J., Meijer E.W. (2000). On the origin of optical activity in polythiophenes. J. Mol. Struct..

[2] Hoeben F.J.M., Jonkheijm P., Meijer E.W., Schenning A.P.H.J. (2005). About supramolecular assemblies of pi-conjugated systems. Chem. Rev..

[3] Kane-Maguire L.A.P., Wallace G.G. (2010). Chiral conducting polymers. Chem. Soc. Rev..

[4] Goto H., Yashima E., Okamoto Y. (2000). Unusual solvent effects on chiroptical properties of an optically active regioregular polythiophene in solution. Chirality.

[5] Goto H., Yashima E. (2002). Electron-induced switching of the supramolecular chirality of optically active polythiophene aggregates. J. Am. Chem. Soc..

[6] Goto H., Okamoto Y., Yashima E. (2002). Solvent-induced chiroptical changes in supramolecular assemblies of an optically active, regioregular polythiophene. Macromolecules.

[7] Leysen P., Teyssandier J., De Feyter S., Koeckelberghs G. (2018). Controlled Synthesis of a Helical Conjugated Polythiophene. Macromolecules.

[8] Liu Y.L., Pauloehrl T., Presolski S.I., Albertazzi L., Palmans A.R.A., Meijer E.W. (2015). Modular Synthetic Platform for the Construction of Functional Single-Chain Polymeric Nanoparticles: From Aqueous Catalysis to Photosensitization. J. Am. Chem. Soc..

[9] Elsabahy M., Heo G.S., Lim S.M., Sun G.R., Wooley K.L. (2015). Polymeric Nanostructures for Imaging and Therapy. Chem. Rev..

[10] O'Reilly R.K., Joralemon M.J., Wooley K.L., Hawker C.J. (2005). Functionalization of micelles and shell cross-linked nanoparticles using click chemistry. Chem. Mater..

[11] Mavila S., Eivgi O., Berkovich I., Lemcoff N.G. (2016). Intramolecular Cross-Linking Methodologies for the Synthesis of Polymer Nanoparticles. Chem. Rev..

[12] Gois J.R., Popov A.V., Guliashvili T., Serra A.C., Coelho J.F.J. (2015). Synthesis of functionalized poly(vinyl acetate) mediated by alkyne-terminated RAFT agents. RSC Adv..

[13] Vandeleene S., Jivanescu M., Stesmans A., Cuppens J., Van Bael M.J., Verbiest T., Koeckelberghs G. (2011). Influence of the Supramolecular Organization on the Magnetic Properties of Poly(3-alkylthiophene)s in Their Neutral State. Macromolecules.

[14] Hammer B.A.G., Bokel F.A., Hayward R.C., Emrick T. (2011). Cross-Linked Conjugated Polymer Fibrils: Robust Nanowires from Functional Polythiophene Diblock Copolymers. Chem. Mater..

[15] Iovu M.C., Jeffries-El M., Sheina E.E., Cooper J.R., McCullough R.D. (2005). Regioregular poly(3-alkylthiophene) conducting block copolymers. Polymer.

[16] Verheyen L., Leysen P., Van den Eede M.P., Ceunen W., Hardeman T., Koeckelberghs G. (2017). Advances in the controlled polymerization of conjugated polymers. Polymer.

[17] Van Camp W., Germonpre V., Mespouille L., Dubois P., Goethals E.J., Du Prez F.E. (2007). New poly(acrylic acid) containing segmented copolymer structures by combination of “Click” chemistry and atom transfer radical polymerization. React. Funct. Polym..

[18] Craley C.R., Zhang R., Kowalewski T., McCullough R.D., Stefan M.C. (2009). Regioregular Poly(3-hexylthiophene) in a Novel Conducting Amphiphilic Block Copolymer. Macromol. Rapid Commun..

[19] Jeffries-El M., Sauve G., McCullough R.D. (2005). Facile synthesis of end-functionalized regioregular poly(3-alkylthiophene)s via modified Grignard metathesis reaction. Macromolecules.

[20] Miyakoshi R., Yokoyama A., Yokozawa T. (2005). Catalyst-transfer polycondensation. Mechanism of Ni-catalyzed chain-growth polymerization leading to well-defined poly(3-hexylthiophene). J. Am. Chem. Soc..

[21] Kamigaito M., Ando T., Sawamoto M. (2001). Metal-Catalyzed Living Radical Polymerization. Chem. Rev..

[22] Matyjaszewski K., Xia J. (2001). Atom Transfer Radical Polymerization. Chem. Rev..

[23] Huisgen R. (1968). Cycloadditions—Definition, Classification, and Characterization. Angew.Chem. Int. Ed. Engl..

[24] Kolb Hartmuth C., Finn M.G., Sharpless K.B. (2001). Click Chemistry: Diverse Chemical Function from a Few Good Reactions. Angew. Chem. Int. Ed..

[25] Binder Wolfgang H., Sachsenhofer R. (2007). ‘Click’ Chemistry in Polymer and Materials Science. Macromol. Rapid Commun..

[26] Satyanarayama T., Abraham S., Kagan H.B. (2009). Nonlinear Effects in Asymmetric Catalysis. Angew. Chem. Int. Ed..

[27] Langeveld-Voss B.M.W., Waterval R.J.M., Janssen R.A.J., Meijer E.W. (1999). Principles of “majority rules” and “sergeants and soldiers” applied to the aggregation of optically active polythiophenes: Evidence for a multichain phenomenon. Macromolecules.

[28] Mahadevi A.S., Sastry G.N. (2016). Cooperativity in Noncovalent Interactions. Chem. Rev..

[29] Cheng C., Qi K., Khoshdel E., Wooley K.L. (2006). Tandem synthesis of core-shell brush copolymers and their transformation to peripherally cross-linked and hollowed nanostructures. J. Am. Chem. Soc..

[30] Huang H.Y., Kowalewski T., Remsen E.E., Gertzmann R., Wooley K.L. (1997). Hydrogel-coated glassy nanospheres: A novel method for the synthesis of shell cross-linked knedels. J. Am. Chem. Soc..

