# Supplementary material for: Synthesis of Amphiphilic Block Copolymers Containing Chiral Polythiophene Chains and Their Micelle Formation and Chiroptical Properties
Source: Polymers (Basel). 2018 Jun 30;10(7):718. doi: 10.3390/polym10070718 (PMC6404056; doi:10.3390/polym10070718)
Supplement: Supplementary file 1 [file polymers-10-00718-s001.pdf]

Supplementary Information

Synthesis of Amphiphilic Block Copolymers Containing Chiral Polythiophene Chains and Their Micelle Formation and Chiroptical Properties

Daisuke Hirose [1], Satoru Nozaki [1], Shigeyoshi Kanoh [1], and Katsuhiro Maeda [1,2*]

1 Graduate School of Natural Science and Technology, Kanazawa University, Kakuma-machi, Kanazawa 920-1192, Japan; dhirose@se.kanazawa-u.ac.jp (D.H.); satoru3959@yahoo.co.jp (S.N.); kanoh@se.kanazawa-u.ac.jp (S.K.)

2 Nano Life Science Institute (WPI-NanoLSI), Kanazawa University, Kakuma-machi, Kanazawa 920-1192, Japan

* Correspondence: maeda@se.kanazawa-u.ac.jp; Tel.: +81-76-234-4783

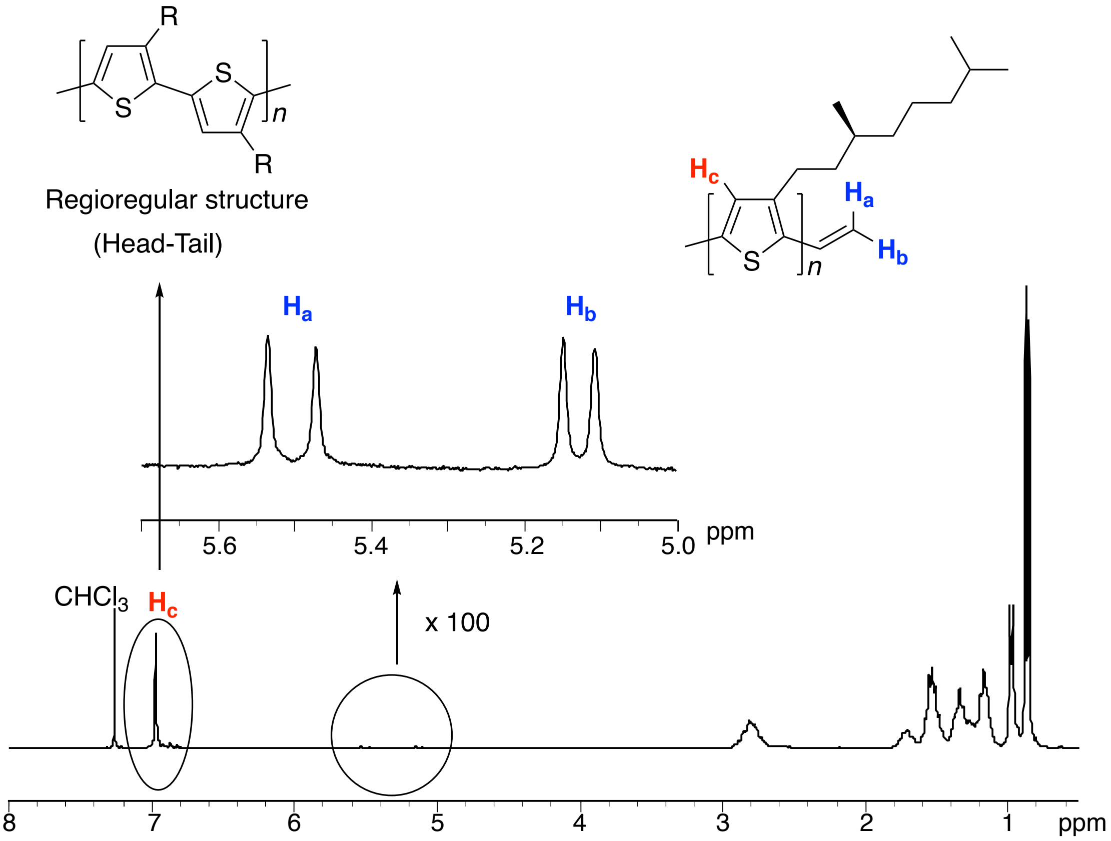

Figure S1. ^{1}H NMR spectrum of (*S*)-poly-**1a** in $CDCl_3$ at rt.

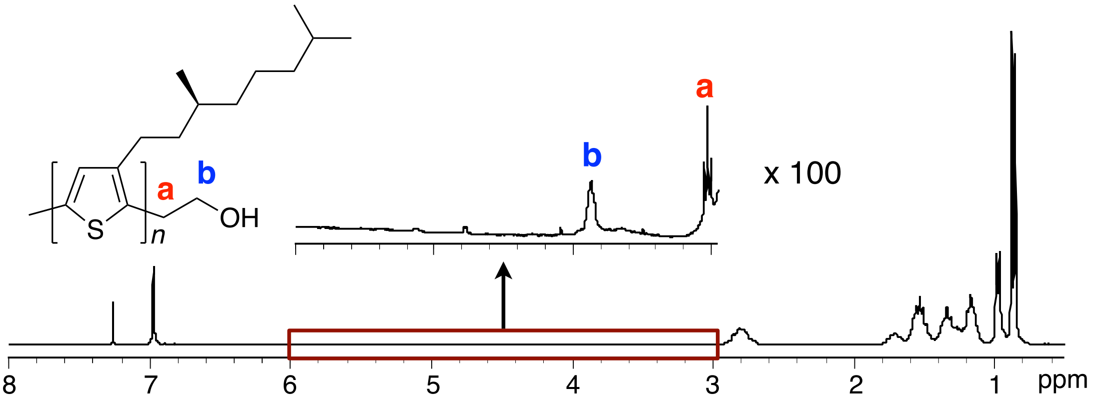

Figure S2. ^{1}H NMR spectrum of (*S*)-poly-**1a**-OH in $CDCl_3$ at rt

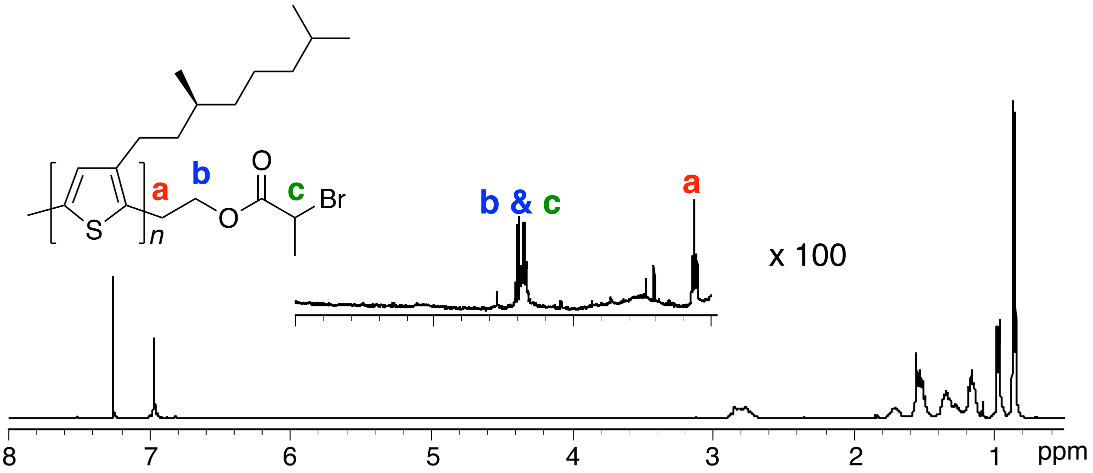

Figure S3. ^{1}H NMR spectrum of (*S*)-poly-**1a**-Br in $CDCl_3$ at rt.

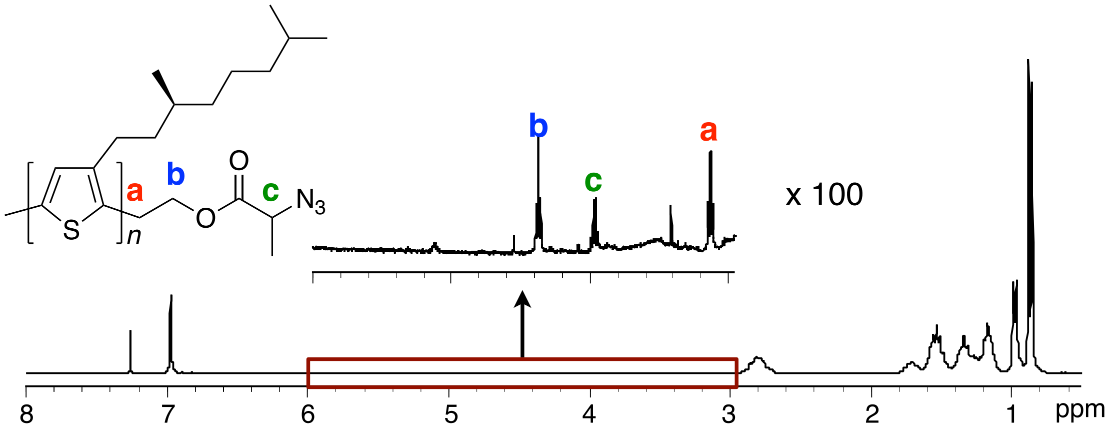

Figure S4. ^{1}H NMR spectrum of (*S*)-poly-**1a**-N_3 in $CDCl_3$ at rt.

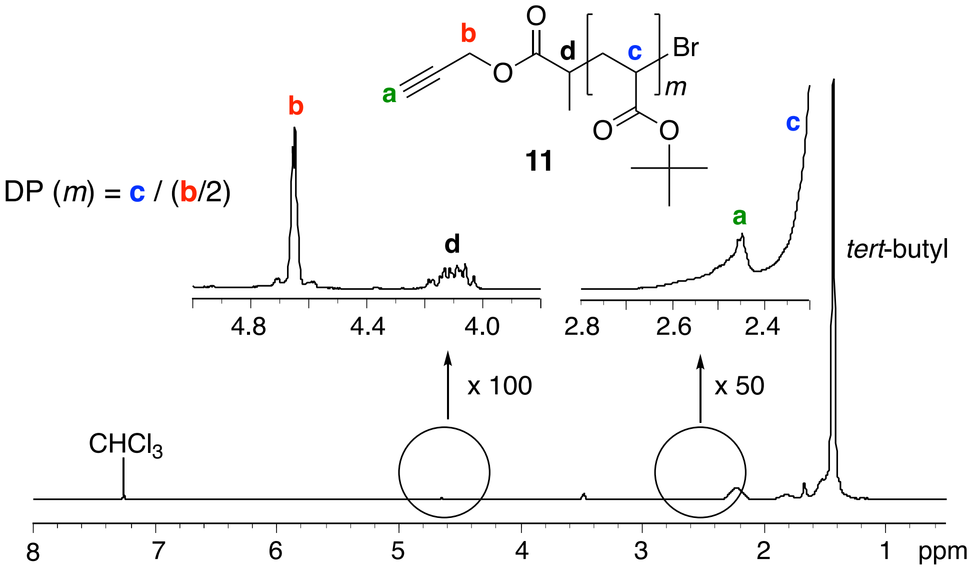

Figure S5. ^{1}H NMR spectrum of poly-**2** in $CDCl_3$ at rt.

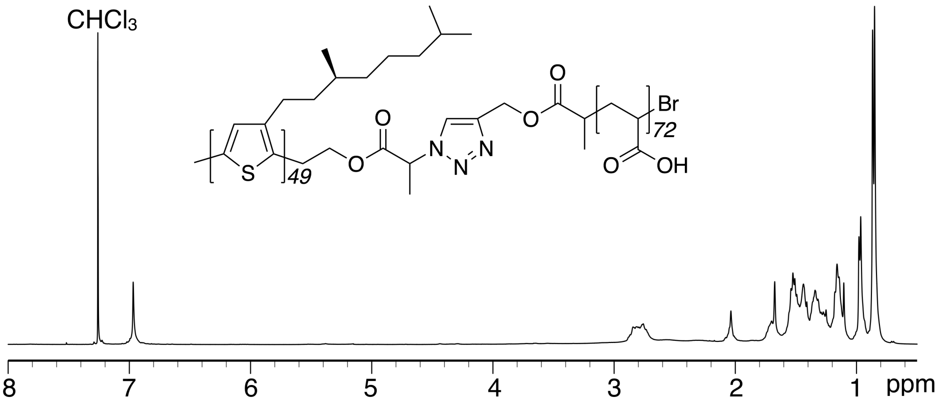

Figure S6. ^{1}H NMR spectrum of (*S*)-poly-**1a**-*b*-poly-**3** in $CDCl_3$ at rt.

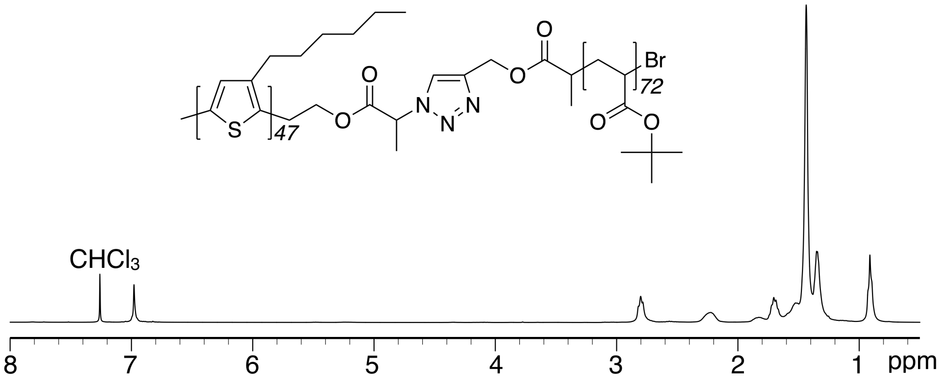

Figure S7. ^{1}H NMR spectrum of poly-**1b**-*b*-poly-**2** in $CDCl_3$ at rt.

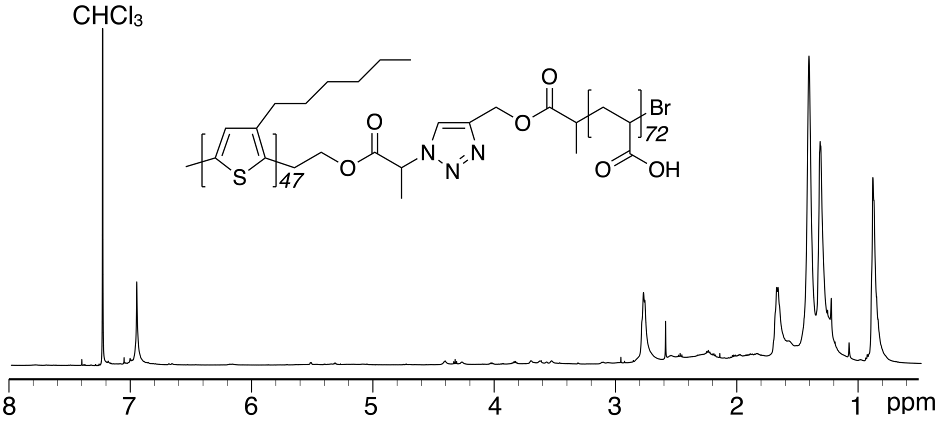

Figure S8. ^{1}H NMR spectrum of poly-**1b**-*b*-poly-**3** in $CDCl_3$ at rt.

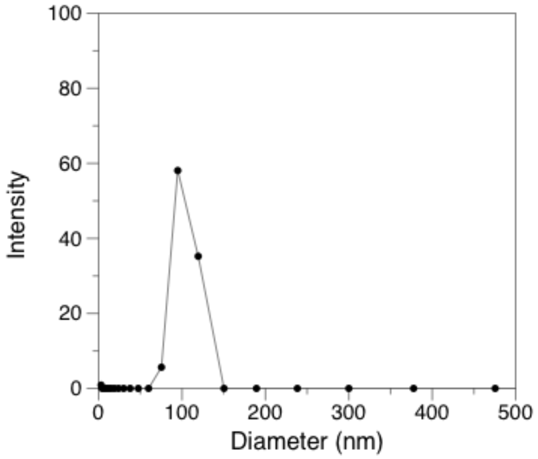

Figure S9. DLS data of the micelles comprising (*S*)-poly-**1a**-*b*-poly-**3** in water at 25 °C.

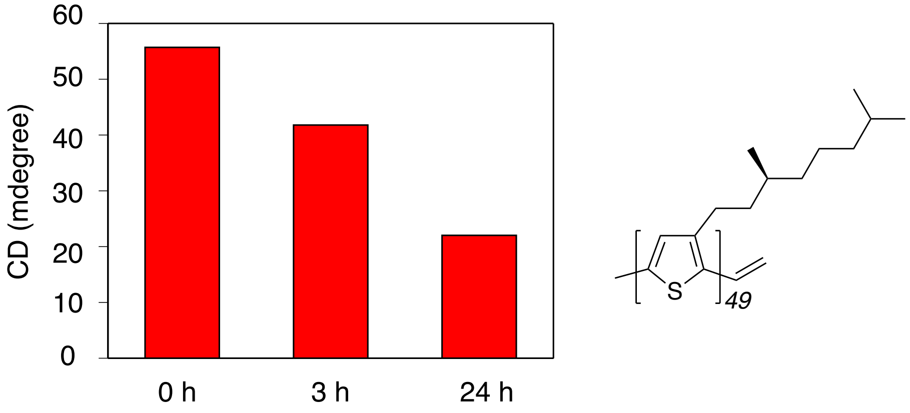

Figure S10. Time-dependent change of CD intensity at 566 nm of (*S*)-poly-**1a** in $CHCl_3$/methanol (1/1, v/v) at rt. [(*S*)-poly-**1a**] = 0.03 mg/mL.
